# Effects of extending interval between presynchronization and breeding protocols and prostaglandin F2α administration before breeding protocol on reproductive performance of Holstein dairy cows during summer

**DOI:** 10.1016/j.vas.2026.100780

**Published:** 2026-07-24

**Authors:** Reza Hemmati Baghbanani, Emadeddin Mobedi, Amir Abbas Mohieddini, Poorya Pooladzadeh, Shayan Zarabadipour, Mehdi Vojgani, Faramarz Gharagozlou, Vahid Akbarinejad

**Affiliations:** a Department of Theriogenology, Faculty of Veterinary Medicine, University of Tehran, Tehran, Iran; b Private Bovine Practitioner, Tehran, Iran

**Keywords:** Synchronization of estrus, Size of follicle, Progesterone concentration, Fertility, Heat stress

## Abstract

Small size of follicle, which may be due to summer, and high progesterone (P4) could hamper the ovulatory response to gonadotropin-releasing hormone (GnRH) at the beginning of GnRH-based protocols for estrus synchronization (GnRH-1), thus decreasing fertility. Accordingly, the objective of this study was to evaluate the effects of two-day extension in interval between presynchronization (Ovsynch) and breeding (Heatsynch) protocols and administration of prostaglandin F2α (PGF_2α_) two days before breeding protocol on size of the largest follicle (LF) and P4 concentration (PC) at GnRH-1, respectively, and fertility of cows during summer. Cows were randomly assigned to four experimental groups, including: 1) O7DH (cows with 7-day interval between Ovsynch and Heatsynch; n = 153), 2) O7DH+PG (cows with 7-day interval between Ovsynch and Heatsynch plus administration of PGF_2α_ two days before Heatsynch; n = 156), 3) O9DH (cows with 9-day interval between Ovsynch and Heatsynch; n = 147), and 4) O9DH+PG (cows with 9-day interval between Ovsynch and Heatsynch plus administration of PGF_2α_ two days before Heatsynch; n = 154). Further, cows were subjected to ultrasonographic examination and blood sampling at GnRH-1. Extension of the interval between Ovsynch and Heatsynch did not affect size of LF at GnRH-1 (P > 0.05); however, it decreased pregnancy risk (P < 0.05). Administration of PGF_2α_ two days before Heatsynch decreased PC at GnRH-1 (P < 0.05), but it did not affect pregnancy risk (P > 0.05). In conclusion, the present study revealed that additional two days before breeding protocol did not increase follicle size at GnRH-1 and reduced fertility. Furthermore, PGF_2α_ administration before breeding protocol could not improve fertility of cows despite reducing PC at GnRH-1.

## Highlights

• Extension in interval between Ovsynch (presynchronization protocol) and Heatsynch (breeding protocol) did not affect size of follicle at GnRH-1.
• Extension in interval between Ovsynch and Heatsynch diminished fertility.
• PGF2α administration before Heatsynch decreased progesterone concentration at GnRH-1.
• PGF2α administration before Heatsynch did not impact fertility.

## Introduction

Application of protocols for synchronization of estrus is an indispensable part of reproductive management in modern dairy herds (Wiltbank & Pursley, 2014). The popularity of these protocols is due to the risk of irregular estrous cycle, ovulation failure and poor behavioral estrus expression in high yielding cows since these factors can hamper timely pregnancy of postpartum cows (Wiltbank & Pursley, 2014). Hence, tremendous efforts have been made to optimize various protocols for synchronization of estrus in cows (Wiltbank & Pursley, 2014). Yet more studies are required to expand our knowledge about factors affecting the efficiency of these protocols in order to understand the potential modifications for improving reproductive programs in dairy herds (Stevenson, 2025; Wiltbank & Pursley, 2014).

One of the important components of GnRH-based protocols (e.g., Ovsynch and Heatsynch) is the responsiveness of cows to the GnRH that initiates the protocol (GnRH-1) (Stevenson, 2025; Wiltbank & Pursley, 2014). Because GnRH-1 is supposed to induce ovulation in the follicle present on the ovary so as to facilitate emergence of a new follicular wave at a predictable timeframe (Stevenson, 2025; Wiltbank & Pursley, 2014). Therefore, the new follicular wave can be managed by further hormonal administrations to achieve synchronized estrus and timed artificial insemination (TAI) (Stevenson, 2025; Wiltbank & Pursley, 2014). As a result, if GnRH-1 fails to remove the follicle prevailing on the ovary, the efficacy of protocol in synchronizing estrus and, in turn, the resulting fertility of cows will decrease (Stevenson, 2025; Wiltbank & Pursley, 2014). In this context, a number of factors have been identified to impact the success of GnRH-1 (Stevenson, 2025; Wiltbank & Pursley, 2014). One of these factors is the dose of GnRH as higher doses (200 μg vs 100 μg) have been shown to increase the likelihood of ovulation following GnRH (Giordano et al., 2013; Valdés-Arciniega et al., 2023). The other factor is the size of the follicle on the ovary as follicles < 10 mm do not show a favorable ovulatory response to GnRH (Keskin et al., 2010; Sartori et al., 2001; Stevenson, 2025). Additionally, high concentrations of P4 could reduce the ovulatory response of follicle to GnRH (Carvalho et al., 2015; Giordano et al., 2012; Stevenson & Pulley, 2016).

Another factor negatively affecting fertility in estrus-synchronized cows is heat stress (Akbarinejad et al., 2017; Emadi et al., 2014; Zarabadipour et al., 2025). More recently, it has been found that summer could have a negative effect on the proportion of animals with follicle ≥ 10 mm at the commencement of breeding protocol in cows that had been presynchronized by Ovsynch (Allahyari et al., 2023). This negative effect of summer was attributed to adverse impacts of heat stress on follicular growth (Allahyari et al., 2023). Because heat stress can decrease follicular growth (Jitjumnong et al., 2020; Schüller et al., 2017) via disrupting gonadotropin secretion, ovarian vascular perfusion, gonadotropin receptor expression, granulosa cell function and steroidogenesis (De Rensis et al., 2021; Nascimento et al., 2025; Wolfenson & Roth, 2019). In this context, limited information was available about how the problem with follicle size at the beginning of breeding protocol during summer could be resolved by modifying the protocols (Allahyari et al., 2023). We hypothesized that extension of interval between presynchronization and breeding protocols may provide extra time for the follicle to grow more and reach the threshold size for more successful effect of GnRH-1 (i.e., ≥ 10 mm) (Keskin et al., 2010; Sartori et al., 2001; Stevenson, 2025). Concomitantly, we assumed that this extension between presynchronization and breeding protocols would provide additional time for the corpus luteum (CL) to develop further, which could result in higher PC at GnRH-1 (Kelley et al., 2009). This possible higher PC could disrupt the potential effect of extension between presynchronization and breeding protocols on follicle size. Because, as aforementioned, while larger follicle size might improve the success of GnRH-1 (Keskin et al., 2010; Sartori et al., 2001; Stevenson, 2025), higher PC could diminish it (Carvalho et al., 2015; Giordano et al., 2012; Stevenson & Pulley, 2016). Besides, it is noteworthy that cows under summer heat stress are less responsive to exogenous GnRH (Garcia-Ispierto et al., 2019; Roth et al., 2021), which could be due to the adverse effects of heat stress on LH secretion and LH receptor expression (De Rensis et al., 2021; Nascimento et al., 2025; Wolfenson & Roth, 2019). Hence, we presumed that higher PC in cows with extended interval between presynchronization and breeding protocols would exacerbate less responsiveness of cows to GnRH, thereby negatively influencing estrus synchronization of cows. In this sense, we hypothesized that PGF_2α_ administration prior to breeding protocol could resolve the issue regarding higher PC (Carvalho et al., 2015). Therefore, we conducted this study to examine the effects of two-day extension between presynchronization (Ovsynch) and breeding (Heatsynch) protocols and PGF_2α_ administration two days before breeding protocol on LF size and PC at GnRH-1 and on the resultant pregnancy risk and loss in Holstein dairy cows during summer.

## Materials and methods

### Animals and locations of the study

Animal Ethics Committee at University of Tehran approved the current research in terms of animal welfare and ethics (IR.UT.VETMED.REC.1403.054). This research was conducted in three commercial Holstein dairy herds (DHs), all of which were located in semi-arid regions and were coded as DH1 (Tehran, Iran; latitude: 35.44°N; longitude: 51.70° E; altitude: 1033 m), DH2 (Qazvin, Iran; latitude: 36.13°N; longitude: 50.17° E; altitude: 1210 m), DH3 (Qazvin, Iran; latitude: 36.01°N; longitude: 50.57° E; altitude: 1211 m). The number of cows was 1500, 3600 and 8200 in herds DH1, DH2 and DH3, respectively. Feeding with a total mixed ration formulated to meet the requirements for lactation (National Academies of Sciences et al., 2021) and milking were performed thrice a day in these herds. Cows were subjected to TAI programs for the first service postpartum, which initiated from day 38 after calving. Cows were examined for pregnancy diagnosis 30–35 days after artificial insemination (AI) and the cows diagnosed pregnant at this stage were re-examined 55–60 days after AI for confirmation of pregnancy. Diagnosis of pregnancy was performed using transrectal ultrasound examination, and all examinations were performed by the same operator. Ultrasonographic examinations were performed using a RKU10 ultrasound machine equipped with a 7.5 MHz rectal linear probe (Kaixin-bio.vet, China) in herds DH1 and DH2 and using an iScan2 ultrasound machine equipped with a 7.5 MHz rectal linear probe (Dramiński S.A., Poland) in herd DH3.

This research was carried out during summer of 2024, and meteorological data related to the locations of the herds were retrieved from https://open-meteo.com. In herd DH1, average temperature, relative humidity, rainfall and wind speed were 32.40 °C, 17.90 %, 0.00 mm and 10.00 km/h, respectively. In herd DH2, average temperature, relative humidity, rainfall and wind speed were 27.41 °C, 37.93 %, 0.00 mm and 9.05 km/h, respectively. In herd DH3, average temperature, relative humidity, rainfall and wind speed were 28.43 °C, 32.70 %, 0.00 mm and 8.75 km/h, respectively. For calculation of temperature humidity index (THI), the formula suggested by Yousef (Yousef, 1985) was used as follows: THI = dry bulb temperature + (0.36 – dew point temperature) + 41.2. Temperature humidity index ≥ 68 was considered as the threshold for heat stress condition (Zimbelman et al., 2009). Based on this THI dataset, environmental condition on days of the study were classified into two THI categories, including mild (THI ≤ 72) and moderate (72 < THI ≤ 80) (Makiabadi et al., 2023).

### Experimental design

**Fig. 1:**
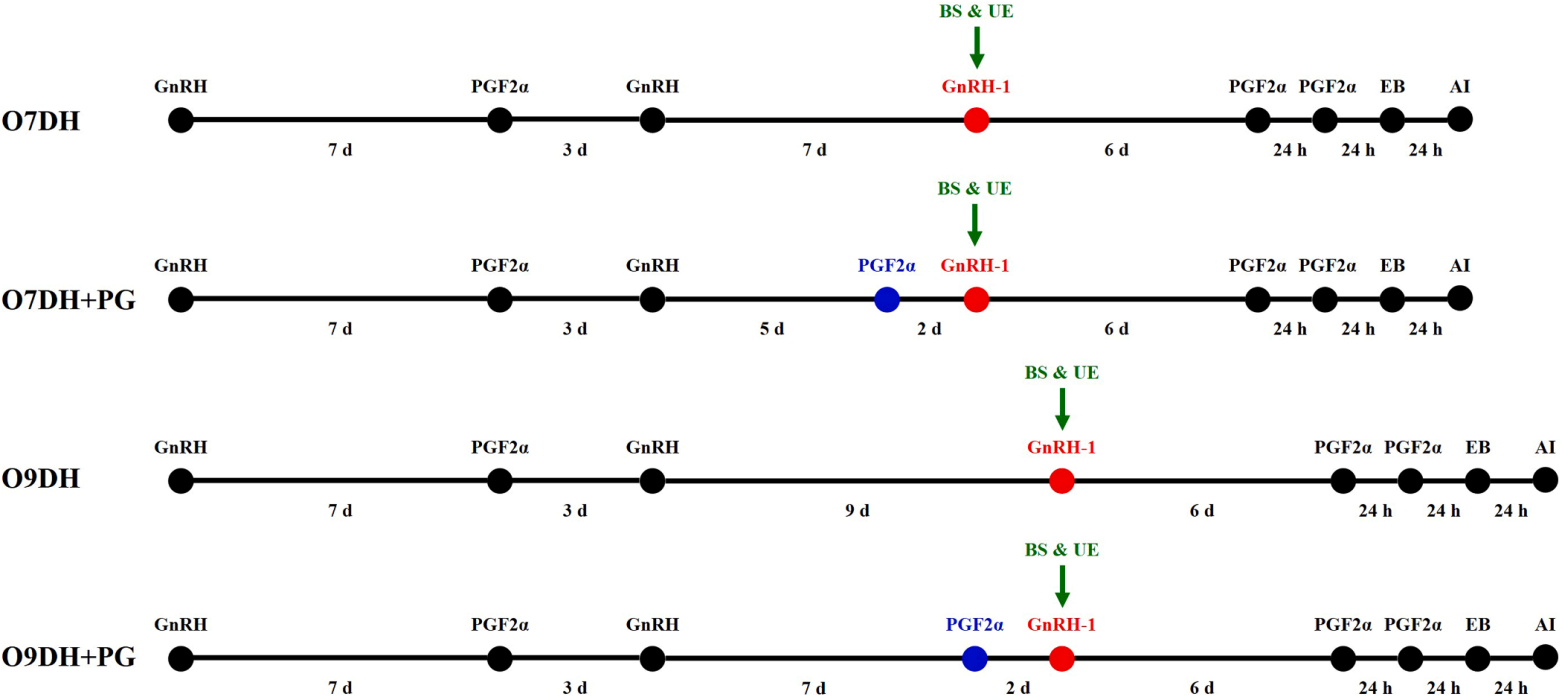
Schematic diagram of hormonal treatments in the present study, in which Ovsynch and Heatsynch were used as presynchronization and breeding protocols, respectively. There were four experimental groups in the present study, including: 1) O7DH (cows with 7-day interval between Ovsynch and Heatsynch), 2) O7DH+PG (cows with 7-day interval between Ovsynch and Heatsynch and with administration of PGF_2α_ two days before Heatsynch, which is highlighted in blue color), 3) O9DH (cows with 9-day interval between Ovsynch and Heatsynch), and 4) O9DH+PG (cows with 9-day interval between Ovsynch and Heatsynch and with administration of PGF_2α_ two days before Heatsynch, which is highlighted in blue color). The protocols were initiated from day 38 postpartum in all experimental groups. Blood sampling (BS) for evaluation of PC and transrectal ultrasonographic examination (UE) for measurement of size of LF were implemented at the time of GnRH administration with which Heatsynch initiated (GnRH-1), which is highlighted in red color.

The study had a 2 × 2 factorial design, by which the main and interaction effects of two-day extension between presynchronization (Ovsynch) and breeding (Heatsynch) protocols and PGF_2α_ administration two days before breeding protocol were evaluated on LF size and PC at GnRH-1 of Heatsynch as well as on consequent pregnancy risk and loss. The inclusion criteria were that cows were required to be free from disorders such as dystocia, retained fetal membrane, metritis, clinical ketosis, abomasal displacement and laminitis. There were four experimental groups including: 1) O7DH (cows with 7-day interval between Ovsynch and Heatsynch; n = 153), 2) O7DH+PG (cows with 7-day interval between Ovsynch and Heatsynch and PGF_2α_ administered two days before Heatsynch; n = 156), 3) O9DH (cows with 9-day interval between Ovsynch and Heatsynch; n = 147), and 4) O9DH+PG (cows with 9-day interval between Ovsynch and Heatsynch and PGF_2α_ administered two days before Heatsynch; n = 154). In this context, all cows were presynchronized with Ovsynch starting on day 38, and received GnRH (100 μg of gonadorelin; Aburaihan Co, Tehran, Iran) followed by PGF_2α_ (500 μg of cloprostenol; Aburaihan Co, Tehran, Iran) and GnRH (100 μg) seven and 10 days later, respectively. Breeding protocol was modified Heatsynch in all cows, which initiated with an injection of GnRH (GnRH-1; 200 μg of gonadorelin) followed by two injections of PGF_2α_ (500 μg of cloprostenol) six and seven days after GnRH-1. Hormonal treatment was finished with an injection of estradiol benzoate (EB; 1 mg; Aburaihan Co, Tehran, Iran) nine days after GnRH-1. Subsequently, the cows were submitted to TAI one day (24 h) after administration of EB. The commencement of these protocols (i.e., presynchronization Ovsynch) was 38 postpartum in all cows; as a result, all experimental groups were consistent with respect to days in milk (DIM) at treatment initiation. The schematic diagram of hormonal treatments in various experimental groups is illustrated in Fig. 1. In this context, 43, 122 and 445 cows (primiparous and multiparous) from herds DH1, DH2 and DH3, respectively, were randomly assigned to the experimental groups of this study (n = 610). In O7DH group, 12, 31 and 110 cows were form herds DH1, DH2 and DH3, respectively. In O7DH+PG group, 11, 33 and 112 cows were form herds DH1, DH2 and DH3, respectively. In O9DH group, 10, 25 and 112 cows were form herds DH1, DH2 and DH3, respectively. In O9DH+PG group, 10, 33 and 111 cows were form herds DH1, DH2 and DH3, respectively. The mean ± SEM of parity was 2.34 ± 0.11, 2.35 ± 0.10, 2.35 ± 0.15 and 2.35 ± 0.12 in O7DH, O7DH+PG, O9DH and O9DH+PG groups, respectively, and it was comparable among various experimental groups (P > 0.05). Herein, it is important to mention that some cows were excluded from the experimental groups over the course of the study due to health issues. As a result, there were 577 cows at GnRH-1 for ultrasonographic examination and blood sampling, including 141, 149, 141 and 146 cows in O7DH, O7DH+PG, O9DH and O9DH+PG groups, respectively. Further, there were 538 cows at the time of pregnancy diagnosis, including 134, 133, 134 and 137 cows in O7DH, O7DH+PG, O9DH and O9DH+PG groups, respectively.

### Ultrasonographic examination

Cows were subjected to ultrasonographic examination of the ovaries at GnRH-1 to measure size of LF at the commencement of breeding protocol (Fig. 1). Ultrasonographic examination was implemented using a RKU10 ultrasound machine equipped with a 7.5 MHz rectal linear probe (Kaixin-bio.vet, China) in herds DH1 and DH2 and using an iScan2 ultrasound machine equipped with a 7.5 MHz rectal linear probe (Dramiński S.A., Poland) in herd DH3. All ultrasonographic examinations were performed by the same operator over the course of the study. Based on LF size, cows were categorized in three classes including LF1 (cows with < 10 mm LF), LF2 (cows with ≥ 10 mm and < 20 mm LF) and LF3 (cows with ≥ 20 mm). The reason for three classes of LF was that follicles < 10 mm do not have an acceptable ovulatory response to GnRH (Keskin et al., 2010; Sartori et al., 2001; Stevenson, 2025). On the other hand, 20 mm has been suggested as the least follicular diameter for ovarian cysts, which might not be as responsive to GnRH as healthy ovarian follicles (Borş & Borş, 2020; Turner et al., 2023). Herein, it is worth mentioning that earlier works frequently used diameter of 25 mm for diagnosis of ovarian cyst (López-Gatius, F. & López-Béjar, 2002; Borş & Borş, 2020; Turner et al., 2023), but the least threshold diameter for ovarian cysts by later studies was considered 20 mm (Borş & Borş, 2020; Turner et al., 2023), which was used in the present study. Moreover, it is notable that only follicular dimeter cannot be considered as a criterion for definitive diagnosis of ovarian cyst, and persistence of large follicle in addition to absence of corpus luteum are necessary conditions for consideration of a follicle larger than 20 mm as ovarian cyst. In this study, we did not intend to study ovarian cysts; therefore, we did not follow up cases with follicles larger than 20 mm to evaluate follicular persistence, and in turn, we could not differentiate whether they were cystic or not. Indeed, by considering follicles larger than 20 mm as a separate category, we simply intended to have a category of follicles in which diameter was larger than the optimal range for an ovulatory follicle (i.e., 10 to 20 mm).

### P4 assay

Blood samples for evaluation of P4 were collected using venipunctures via the coccygeal vein at GnRH-1 to determine PC at the commencement of breeding protocol (Fig. 1). The samples were centrifuged (15 min at 2000 × g) within 2 h after collection, and the sera were stored at −20 °C until P4 assay. PC was assessed using a commercial kit (Progesterone ELISA Kit, Ideal Tashkhis Atieh Company, Karaj, Iran) based on the manufacturer’s instructions (detection limit: 0.12 ng/mL; intra-assay coefficient of variation: 4.3 %; inter-assay coefficient of variation: 4.9 %). Based on PC, cows were categorized in three classes including PC1 (cows with < 0.5 ng/mL PC), PC2 (cows with ≥ 0.5 ng/mL and < 2.0 ng/mL PC) and PC3 (cows with ≥ 2.0 ng/mL PC). The reason for three classes of PC was that PC1, PC2 and PC3 are considered as low, suprabasal and high PC, which can differentially affect response to GnRH and LH secretion (Robinson et al., 2006; Carvalho et al., 2015; Giordano et al., 2012; Stevenson & Pulley, 2016).

### Reproductive indices

For calculation of pregnancy per AI (P/AI), the number of cows diagnosed pregnant was divided by the number of cows submitted to TAI, which was further multiplied by 100. For calculation of pregnancy loss, the number of cows that lost their pregnancy between the first and second ultrasonographic examinations for pregnancy diagnosis was divided by the number of cows diagnosed pregnant at the first ultrasonographic examination, which was further multiplied by 100.

### Statistical analysis

In addition to data of experimental groups and THI, data associated with average daily milk production of the first three months postpartum of each individual cow were retrieved from the database of the herds since level of milk production can influence reproduction in dairy animals (Akbarinejad & Cushman, 2024). Data of average milk production were standardized using Z-score method before being used in statistical analysis as follows:

$$z\;=\;\frac{x-\;\mu }{\sigma }$$

Where:

*z* = the standard score (Z-score)

*x* = the raw data point

*σ* = the standard deviation of population

Further, data of size of LF and PC were tested for normality using Kolmogorov-Smirnov test (UNIVARIATE procedure). Data of PC were positively skewed and were subjected to log-plus-one transformation before analysis as follows:

$$y^{\prime }\;=\;\text{ln}\left(x+1\right)$$

Where:

*y′* = transformed data

*x* = raw data

Data of LF and PC were analyzed using GLM procedure, and data associated with proportion of cows in different categories of LF and PC were analyzed using GENMOD procedure (logistic regression), in which the distribution of the response variables was considered binomial and logit link function was included. In the analyses conducted using GLM and GENMOD procedures, interaction effect of interval between presynchronization and breeding protocols by PGF_2α_ administration two days before breeding protocol as well as their main effects, parity, THI category at GnRH-1, herd ID and average daily milk production of the first three months postpartum (as a covariate) were included as fixed effects in the model. Fixed effects were evaluated using Type III F-tests. The model for analysis of LF and PC was as follows:

$$y_{ijklmn}=\mu +I_{i}+PG_{j}+{\left(I\times PG\right)}_{ij}+PA_{k}+THI_{l}+DH_{m}+\gamma M_{ijklmn}+\varepsilon_{ijklmn}$$

*y_ijklmn_* = dependent variable in interval group i, PGF_2α_ group j, parity category k, THI category l, herd ID m, and with average milk production for the specific observation n within the group defined by i, j, k, l and m

Where:

*μ* = overall mean

*I_i_* = fixed main effect of interval group i

*PG_j_* = fixed main effect of PGF_2α_ group j

*(I × PG)_ij_* = fixed interaction effect of interval group i by PGF_2α_ group j

*PA_k_* = categorical fixed effect of parity k

*THI_l_* = categorical fixed effect of THI category l

*DH_m_* = categorical fixed effect of herd ID m

*γ* = the regression coefficient for the continuous fixed effect of average milk production

*M* = continuous fixed effect of average milk production

*ε_ijklmn_* = random error in interval group i, PGF_2α_ group j, parity category k, THI category l, herd ID m, and with average milk production for the specific observation n within the group defined by i, j, k, l and m

Furthermore, data associated with P/AI and pregnancy loss were analyzed using GENMOD procedure, in which the distribution of the response variables was considered binomial and logit link function was included. In this regard, the main effects of the interval between presynchronization and breeding protocols and PGF_2α_ administration two days before breeding protocol, their interaction effect (i.e., Interval × PGF_2α_), category of LF (CLF), category of PC (CPC), parity, THI category at GnRH-1, herd ID and average daily milk production of the first three months postpartum (as a covariate) were included as fixed effects in the model. Logistic regression analyses generated odds ratio (OR) as a measure for the strength of difference between or among independent variables regarding their impact on dependent variables. Moreover, LSMEANS statement was used to conduct multiple comparisons, in which PDIFF was used to perform pairwise comparisons. All analyses were performed in SAS version 9.4 (SAS Institute Inc., Cary, NC, USA). Visualization of data was implemented using some libraries of Python including Pandas, Matplotlib, Seaborn and SciPy. Data of dependent variables are presented as percentages (binomial variables) or mean ± SEM (continuous variables). Differences at *P* < 0.05 were considered significant, and differences at 0.05 ≤ *P* < 0.10 were considered as tendencies.

## Results

### Climatic data

The mean ± SEM of THI during the period of time this study was conducted was 74.91 ± 0.23, 71.82 ± 0.18 and 72.31 ± 0.19 89 in herds DH1, DH2 and DH3, respectively. In addition, the range of THI during this period was 69.09 to 78.00, 67.42 to 75.32 and 68.32 to 75.89 in herds DH1, DH2 and DH3, respectively. The only day with THI slightly lower than 68, as the threshold for heat stress condition (Zimbelman et al., 2009), was August 17 in herd DH2. It is worth mentioning that on this date in herd DH2, there was no record for administration of GnRH-1 in a cow.

### Size of LF at GnRH-1

**Fig. 2:**
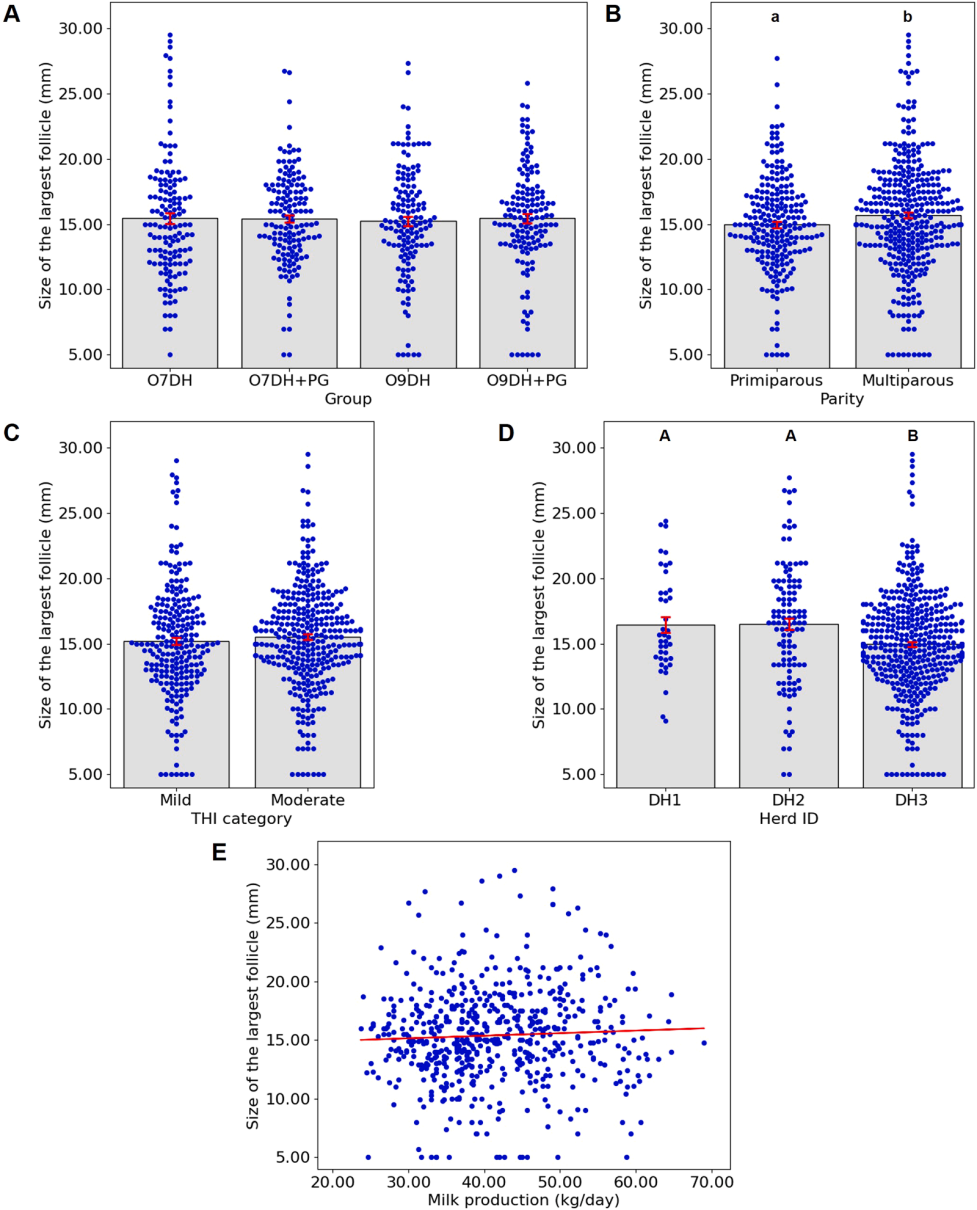
A) Size of LF at GnRH-1 in O7DH (cows with 7-day interval between Ovsynch and Heatsynch; n = 141), O7DH+PG (cows with 7-day interval between Ovsynch and Heatsynch and with administration of PGF_2α_ two days before Heatsynch; n = 149), O9DH (cows with 9-day interval between Ovsynch and Heatsynch; n = 141), and O9DH+PG (cows with 9-day interval between Ovsynch and Heatsynch and with administration of PGF_2α_ two days before Heatsynch; n = 146) groups. B) Size of LF at GnRH-1 in primiparous (n = 226) and multiparous (n = 351) cows. C) Size of LF at GnRH-1 in mild (n = 237) and moderate (n = 340) THI categories. D) Size of LF at GnRH-1 in herds DH1 (n = 40) DH2 (n = 115) and DH3 (n = 422) cows. E) Size of LF at GnRH-1 in relation to average daily milk production during the first three months postpartum (n = 577). Data are presented as mean ± SEM. Various small letters (a,b) denote tendency to significant difference among the specified groups (0.05 ≤ P < 0.10). Various capital letters (A,B) denote significant difference among the specified groups (P < 0.05).

Mean ± SEM (range) of LF size was 15.45 ± 0.40 mm (5.00 to 29.50 mm), 15.41 ± 0.29 mm (5.00 to 26.70 mm), 15.22 ± 0.35 mm (5.00 to 27.30 mm) and 15.44 ± 0.34 mm (5.00 to 25.80 mm) in O7DH, O7DH+PG, O9DH and O9DH+PG groups, respectively. The main and interaction effects of interval and PGF_2α_ administration did not influence LF size (P > 0.05; Fig. 2A). There was a tendency for association of parity with size of LF (*P* = 0.066; Fig. 2B). Moreover, herd differences were observed in size of LF (*P* < 0.05; Fig. 2D). However, LF size was not different between THI categories (P > 0.05; Fig. 2C) and it was not associated with milk production (P > 0.05; Fig. 2E).

PGF_2α_ moderately increased proportion of cows in LF2 category in cows with 7-day interval (OR = 2.051, 95 % CI = 1.075–3.910; *P* = 0.029), but not in cows with 9-day interval (*P* > 0.05; Table 1). Additionally, proportion of cows in LF2 category was greater in primiparous than multiparous cows (OR = 1.844, 95 % CI = 1.037–3.278; *P* = 0.037; Table 1). There was also a tendency for herd differences in proportion of cows in LF2 category (0.05 ≤ *P* < 0.10; Table 1).

**Table 1:** Proportion of cows in various categories of the largest follicle (LF) at GnRH-1 considering experimental group, parity, THI category and herd ID. Data are presented as percentages, and values in parenthesis are actual numbers.

		Category of LF		
Effect	Class	LF1	LF2	LF3
Experimental group	O7DH	9.22 (13/141)	78.72 (111/141)^A^	12.06 (17/141)
	O7DH+PG	4.70 (7/149)	87.92 (132/149)^B^	7.38 (11/149)
	O9DH	9.22 (13/141)	78.72 (111/141)^A^	12.06 (17/141)
	O9DH+PG	10.28 (15/146)	78.08 (114/146)^A^	11.64 (17/146)
				
Parity	Primiparous	7.08 (16/226)	85.40 (193/226)^A^	7.52 (17/226)
	Multiparous	9.12 (32/351)	78.06 (274/351)^B^	12.82 (45/351)
				
THI category	Mild	8.86 (21/237)	80.59 (191/237)	10.55 (25/237)
	Moderate	7.94 (27/340)	81.18 (276/340)	10.88 (37/340)
Herd ID	DH1	5.00 (2/40)	72.50 (29/40)^a^	22.50 (9/40)^A^
	DH2	6.96 (8/115)	74.78 (86/115)^a^	18.26 (21/115)^A^
	DH3	9.01 (38/422)	83.41 (352/422)^b^	7.58 (32/422)^B^

LF1: Cows with LF < 10 mm.

LF2: Cows with LF ≥ 10 mm and < 20 mm.

LF3: Cows with LF ≥ 20 mm.

O7DH: Cows with 7-day interval between Ovsynch and Heatsynch.

O7DH+PG: Cows with 7-day interval between Ovsynch and Heatsynch and with administration of PGF2α two days before Heatsynch.

O9DH: Cows with 9-day interval between Ovsynch and Heatsynch.

O9DH+PG: Cows with 9-day interval between Ovsynch and Heatsynch and with administration of PGF2α two days before Heatsynch.

a,bVarious small letters denote tendency to significant difference among the specified groups (0.05 ≤ P < 0.10).

A,BVarious capital letters denote significant difference among the specified groups (P < 0.05).

Herd differences were also observed in proportion of cows in LF3 category (*P* < 0.01; Table 1).

### Concentration of P4 at GnRH-1

**Fig. 3:**
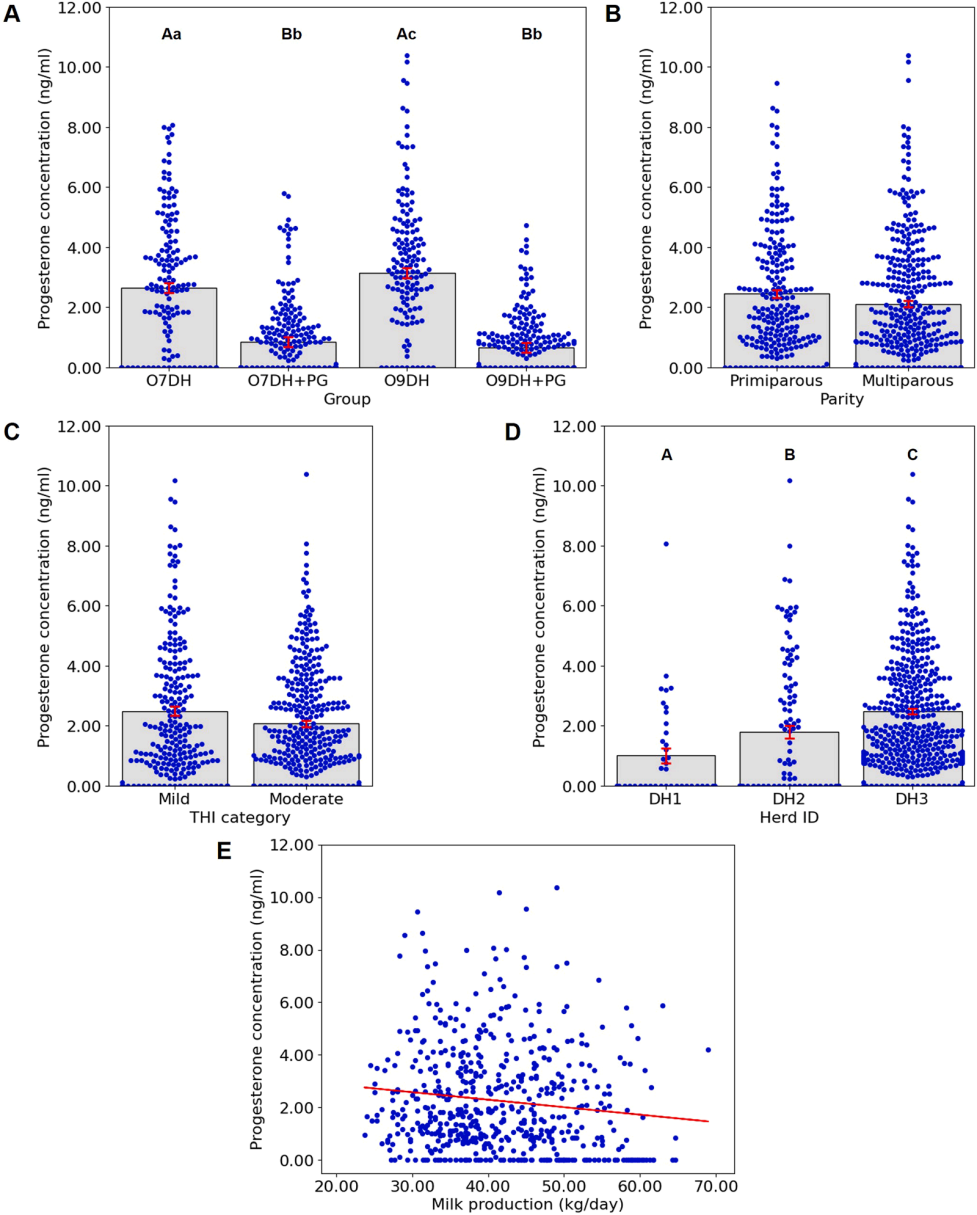
A) PC at GnRH-1 in O7DH (cows with 7-day interval between Ovsynch and Heatsynch; n = 141), O7DH+PG (cows with 7-day interval between Ovsynch and Heatsynch and with administration of PGF_2α_ two days before Heatsynch; n = 149), O9DH (cows with 9-day interval between Ovsynch and Heatsynch; n = 141), and O9DH+PG (cows with 9-day interval between Ovsynch and Heatsynch and with administration of PGF_2α_ two days before Heatsynch; n = 146) groups. B) PC at GnRH-1 in primiparous (n = 226) and multiparous (n = 351) cows. C) PC at GnRH-1 in mild (n = 237) and moderate (n = 340) THI categories. D) PC at GnRH-1 in herds DH1 (n = 40) DH2 (n = 115) and DH3 (n = 422) cows. E) PC at GnRH-1 in relation to average daily milk production during the first three months postpartum (n = 577). Data are presented as mean ± SEM. Various small letters (a,b,c) denote tendency to significant difference among the specified groups (0.05 ≤ P < 0.10). Various capital letters (A,B,C) denote significant difference among the specified groups (P < 0.05).

Mean ± SEM (range) of PC was 3.06 ± 0.18 ng/mL (0.00 to 8.07 ng/mL), 1.27 ± 0.11 ng/mL (0.00 to 5.80 ng/mL), 3.59 ± 0.19 ng/mL (0.00 to 10.38 ng/mL) and 1.11 ± 0.08 ng/mL (0.00 to 4.72 ng/mL) in O7DH, O7DH+PG, O9DH and O9DH+PG groups, respectively. PC was influenced by the interaction effect of interval by PGF_2α_ administration (P = 0.015). In this regard, extension in interval led to a tendency for elevation in PC in PGF_2α_ -untreated cows (*P* = 0.052), but not in PGF_2α_ -treated cows (*P* > 0.05). PGF_2α_ reduced PC regardless of interval (*P* < 0.0001; Fig. 3A). Furthermore, herd differences were observed in PC (*P* < 0.05; Fig. 3D). However, PC was not different between parities (*P* < 0.05; Fig. 3B) and THI categories (*P* < 0.05; Fig. 3C), and it was not associated with milk production (*P* < 0.05; Fig. 3E).

Extension in interval decreased proportion of cows in PC1 category regardless of PGF_2α_ administration (OR = 0.551, 95 % CI = 0.332–0.913; *P* = 0.021), and PGF_2α_ increased this proportion regardless of interval (OR = 3.177, 95 % CI = 1.877–5.375; *P* < 0.0001; Table 2). Moreover, herd differences were observed in proportion cows in PC1 category (*P* < 0.0001; Table 2).

**Table 2:** Proportion of cows in various categories of progesterone concentration (PC) at GnRH-1 considering experimental group, parity, THI category and herd ID. Data are presented as percentages, and values in parenthesis are actual numbers.

			Category of PC	
Effect	Class	PC1	PC2	PC3
Experimental group	O7DH	18.44 (26/141)^A^	14.89 (21/141)^A^	66.67 (94/141)^A^
	O7DH+PG	29.53 (44/149)^B^	52.35 (78/149)^B^	18.12 (27/149)^C^
	O9DH	8.51 (12/141)^C^	13.48 (19/141)^A^	78.01 (110/141)^B^
	O9DH+PG	24.66 (36/146)^AB^	61.64 (90/146)^B^	13.70 (20/146)^C^
Parity	Primiparous	13.27 (30/226)	39.38 (89/226)	47.35 (107/226)
	Multiparous	25.07 (88/351)	33.90 (119/351)	41.03 (144/351)
THI category	Mild	18.14 (43/237)	35.87 (85/237)	45.99 (109/237)
	Moderate	22.06 (75/340)	36.18 (123/340)	41.76 (142/340)
Herd ID	DH1	55.00 (22/40)^A^	22.50 (9/40)^A^	22.50 (9/40)^Aa^
	DH2	51.30 (59/115)^A^	9.57 (11/115)^B^	39.13 (45/115)^Bb^
	DH3	8.77 (37/422)^B^	44.55 (188/422)^C^	46.68 (197/422)^Bc^

PC1: Cows with PC < 0.5 ng/mL.

PC2: Cows with PC ≥ 0.5 ng/mL and < 2.0 ng/mL.

PC3: Cows with PC ≥ 2.0 ng/mL.

O7DH: Cows with 7-day interval between Ovsynch and Heatsynch.

O7DH+PG: Cows with 7-day interval between Ovsynch and Heatsynch and with administration of PGF2α two days before Heatsynch.

O9DH: Cows with 9-day interval between Ovsynch and Heatsynch.

O9DH+PG: Cows with 9-day interval between Ovsynch and Heatsynch and with administration of PGF2α two days before Heatsynch.

a,b,cVarious small letters denote tendency to significant difference among the specified groups (0.05 ≤ P < 0.10).

^A,B,C^Various capital letters denote significant difference among the specified groups (P < 0.05).

PGF_2α_ increased proportion of cows in PC2 category regardless of interval (OR = 10.942, 95 % CI = 7.014–17.070; *P* < 0.0001; Table 2). In addition, milk production was negatively associated with proportion of cows in PC2 category (*P* = 0.048). Herd differences were also observed in proportion cows in PC2 category (P < 0.05; Table 2).

Extension in interval led to increased proportion of cows in PC3 category in PGF_2α_-untreated cows (OR = 1.814, 95 % CI = 1.049–3.138; *P* = 0.033), but not in PGF_2α_-treated cows (*P* > 0.05). PGF_2α_ decreased proportion of cows in PC3 category regardless of interval (*P* < 0.0001; Table 2). Herd differences were also observed in proportion cows in PC3 category (P < 0.05; Table 2).

### Pregnancy per AI and pregnancy loss

Extension in interval decreased pregnancy per AI at the first examination for pregnancy diagnosis (OR = 0.544, 95 % CI = 0.378–0.782; *P* = 0.001; Table 3). Pregnancy per AI at the first examination for pregnancy diagnosis in LF3 category tended to be greater than that in LF1 category (*P* = 0.086; Table 3). In addition, it was greater in primiparous than multiparous cows (OR = 1.714, 95 % CI = 1.049–2.801; *P* = 0.032; Table 3). There were also herd differences in this regard (*P* < 0.05; Table 3).

**Table 3:** Pregnancy per AI (P/AI) at the first and second ultrasonographic examinations for pregnancy diagnosis and pregnancy loss between the first and second examinations considering experimental group, category of size of the largest follicle (LF) at GnRH-1, category of progesterone concertation (PC) at GnRH-1, parity, THI category and herd ID. Data are presented as percentages, and values in parenthesis are actual numbers.

Effect	Class	P/AI at the first examination (%)	P/AI at the second examination (%)	Pregnancy loss (%)
Experimental group	O7DH	44.03 (59/134)^AC^	33.58 (45/134)^ABac^	23.73 (14/59)
	O7DH+PG	45.11 (60/133)^A^	36.84 (49/133)^Aa^	18.33 (11/60)
	O9DH	29.85 (40/134)^B^	23.88 (32/134)^Bb^	20.00 (8/40)
	O9DH+PG	33.58 (46/137)^BC^	23.36 (32/137)^Bbc^	30.43 (14/46)
Category of LF	LF1	27.27 (12/44)^a^	15.91 (7/44)^Aa^	41.67 (5/12)^A^
	LF2	38.71 (168/434)^ab^	29.49 (128/434)^ABa^	23.81 (40/168)^AB^
	LF3	41.67 (25/60)^b^	38.33 (23/60)^Bb^	8.00 (2/25)^B^
Category of PC	PC1	35.71 (40/112)	30.36 (34/112)	15.00 (6/40)
	PC2	38.42 (73/190)	28.95 (55/190)	24.66 (18/73)
	PC3	38.98 (92/236)	29.24 (69/236)	25.00 (23/92)
Parity	Primiparous	43.27 (90/208)^A^	33.17 (69/208)^A^	23.33 (21/90)
	Multiparous	34.85 (115/330)^B^	26.97 (89/330)^B^	22.61 (26/115)
THI category	Mild	35.29 (78/221)	26.70 (59/221)	24.36 (19/78)
	Moderate	40.06 (127/317)	31.23 (99/317)	22.05 (28/127)
Herd ID	DH1	42.50 (17/40)^AB^	40.00 (16/40)	5.88 (1/17)^AB^
	DH2	28.57 (32/112)^A^	26.79 (30/112)	6.25 (2/32)^A^
	DH3	40.41 (156/386)^B^	29.02 (112/386)	28.21 (44/156)^B^

PC1: Cows with PC < 0.5 ng/mL.

PC2: Cows with PC ≥ 0.5 ng/mL and < 2.0 ng/mL.

PC3: Cows with PC ≥ 2.0 ng/mL.

LF1: Cows with LF < 10 mm.

LF2: Cows with LF ≥ 10 mm and < 20 mm.

LF3: Cows with LF ≥ 20 mm.

O7DH: Cows with 7-day interval between Ovsynch and Heatsynch.

O7DH+PG: Cows with 7-day interval between Ovsynch and Heatsynch and with administration of PGF2α two days before Heatsynch.

O9DH: Cows with 9-day interval between Ovsynch and Heatsynch.

O9DH+PG: Cows with 9-day interval between Ovsynch and Heatsynch and with administration of PGF2α two days before Heatsynch.

^a,b,c^Various small letters denote tendency to significant difference among the specified groups (0.05 ≤ P < 0.10).

^A,B,C^Various capital letters denote significant difference among the specified groups (P < 0.05).sath.

Extension in interval decreased pregnancy per AI at the second examination for pregnancy diagnosis (OR = 0.543, 95 % CI = 0.369–0.801; *P* = 0.002; Table 3). Pregnancy per AI at the second examination for pregnancy diagnosis in LF3 category was greater than that in LF1 category (OR = 3.310, 95 % CI = 1.236–8.866; *P* = 0.017), and tended to be greater than that in LF2 category (*P* = 0.082; Table 3). Moreover, it was greater in primiparous than multiparous cows (OR = 1.820, 95 % CI = 1.077–3.076; *P* = 0.025; Table 3).

Pregnancy loss in LF3 category was moderately less than that in LF1 category (OR = 0.140, 95 % CI = 0.020–0.984; *P* = 0.048; Table 3). There were also herd differences in pregnancy loss (*P* < 0.05; Table 3).

## Discussion

The working hypotheses in the present study were that extension in interval between presynchronization and breeding protocols and PGF_2α_ administration two days before breeding protocol could lead to increased size of LF and decreased PC, respectively, at GnRH-1 in cows during summer. In this sense, larger follicle (Keskin et al., 2010; Sartori et al., 2001; Stevenson, 2025) and lower PC (Carvalho et al., 2015; Giordano et al., 2012; Stevenson & Pulley, 2016) were considered as better ovarian and hormonal conditions for commencement of a GnRH-based breeding protocol, which was Heatsynch in the current study.

In this context, the hypothesis on the effect of providing additional time for greater follicular growth was rejected as extension in the interval between presynchronization and breeding protocols did not increase size of LF at GnRH-1. It is notable that proportion of cows with follicles larger than 10 mm in the present study (90.78 %) was substantially greater than that in the previous study (55.00 %) (Allahyari et al., 2023). The adverse effects of heat stress on follicular growth and function is well-established (De Rensis et al., 2021; Jitjumnong et al., 2020; Nascimento et al., 2025; Schüller et al., 2017; Wolfenson & Roth, 2019). For example, Schüller et al. (2017) studied the relationship between heat stress and ovarian structures in estrous cows, and observed that size of follicle declined 0.1 mm per each unit increase in THI at the day of estrus. Moreover, Jitjumnong et al. (2020) investigated cows maintained under tropical conditions by Doppler ultrasonographic examination, and found an inverse association of THI with size and blood flow of preovulatory follicle and estradiol concentration. However, there are other studies indicating no significant effect of heat stress on follicle size (Roth et al., 2001; Vanselow et al., 2016). In this regard, Roth et al. (2001) found no impact of heat stress on diameter of medium-size and preovulatory follicles and estradiol concentration of follicular fluid in cows maintained in environmental chambers. Furthermore, Vanselow et al. (2016) observed no effect of acute heat stress on size of preovulatory folicles in cows maintained in climate/respiration chambers. Besides, it is worth noting that although THI is the most widely used index for evaluation of thermal environment, it is not the only factor determining heat stress intensity and its consequences in cattle (Mylostyvyi & Izhboldina, 2025; Oliveira et al., 2025). Indeed, intensity of heat stress on dairy cows could be influenced by a multitude of factors other than THI, including milk production level (Oliveira et al., 2025), nutritional management (Sammad et al., 2020), air velocity (Gaughan et al., 2023; Oliveira et al., 2025), solar radiation load (Oliveira et al., 2025), nighttime recovery period (Gaughan et al., 2023; Mylostyvyi & Izhboldina, 2025; Oliveira et al., 2025), individual resistance (Zarabadipour et al., 2025) and cooling strategies (Gaughan et al., 2023; Mylostyvyi & Izhboldina, 2025; Oliveira et al., 2025). These factors could be different among various regions and herds, which could lead to herd variations in negative effects of hot summertime and elevated THI on reproductive features such as follicular growth (Jitjumnong et al., 2020; Roth et al., 2001; Schüller et al., 2017; Vanselow et al., 2016). In corroboration with this concept, some minor differences in follicle size were also observed among different herds in the current study. In addition to environmental and herd dissimilarities, different experimental settings of the current study versus the study by Allahyari et al. (2023) could have contributed to different proportion of cows with follicle larger than 10 mm between these two studies. These results accentuate the importance of considering herd physiological conditions when evaluating the effectiveness of protocols for estrus synchronization in cows.

Regardless of underlying mechanisms, these findings implied that follicular growth in the present study was not as impaired during summer as was it in the previous study (Allahyari et al., 2023). Hence, absence of intensely reduced follicular growth in the current study might be one potential reason for ineffectiveness of extended interval between presynchronization and breeding protocols on follicle size. Nonetheless, it does not mean that extended interval could have been necessarily effective if follicular growth had been slowed down during summer. Therefore, further research is required to understand whether this approach could improve efficiency of breeding protocol in cows with reduced follicular growth.

Interestingly, PGF_2α_ administration in cows with 7-day interval (O7DH group vs O7DH+PG group) resulted in a significant increase in proportion of cows in LF2 category and a non-significant decrease in proportion of cows in LF1 category. This phenomenon might have resulted from the decline in PC in response to PGF_2α_. Because P4 inhibits GnRH pulses and LH secretion; therefore, reduction in PC would be followed by elevation in LH secretion (Cerri et al., 2011a, 2011b; e Silva et al., 2025; Stevenson & Pulley, 2016). LH is the primary driver of development in dominant follicles since ovarian follicles switch form FSH-dependent growth to LH-dependent growth after follicular deviation (Gomez-León et al., 2020; Mihm et al., 2006). However, PGF_2α_ administration did not affect follicle size in the cows with 9-day interval (O9DH group vs O9DH+PG group), which could be partly due to two-day later decline in PC in these cows as compared with cows with 7-day interval. Notably, LH-dependent growth of ovarian follicles following follicular deviation continues for a limited period of time, during which follicles either grow (i.e., growing phase) or plateau (i.e., static phase) (Adams et al., 2008; Price & Estienne, 2018). Afterwards, follicles enter a regressing phase, during which they lose their LH receptors, and in turn, elevated LH secretion could not impact their growth anymore (Adams et al., 2008; Price & Estienne, 2018; Xu et al., 1995). In this regard, comparable proportion of cows in LF1, LF2 and LF3 categories in O7DH group vs O9DH and O9DH+PG groups indicates that there was limited dynamics in follicular growth during the additional time between presynchronization and breeding protocols. This phenomenon implies that follicles at GnRH-1 in 9-day interval groups might have been on the verge of entering regressing phase during the extra two days between presynchronization and breeding protocols. It could be partly the reason that PGF_2α_ administration would not change proportion of cows in LF2 category in O9DH+PG group as did it in O7DH+PG group, even though it decreased PC in both O9DH+PG and O7DH+PG groups.

Further, it was revealed that size of LF at GnRH-1 was positively associated with pregnancy risk and negatively associated with pregnancy loss. It has been indicated that fertility of oocytes ovulating from either too small or too large follicles are inferior (Perry et al., 2007, 2005; Vasconcelos et al., 2001, 1999). Yet herein, it is worth noting that we do not expect conception from the follicle at GnRH-1. Alternatively, the follicle prevailing on the ovaries at GnRH-1 should ovulate within approximately 48 h in response to GnRH so that a new follicular wave could emerge on the ovaries. Indeed, the timepoints for further hormonal treatments of breeding protocol are based on the assumption that ovulatory response to GnRH-1 facilitated emergence of a new follicular wave at a favorable timeframe. Otherwise, further hormonal treatments of protocol may not be reasonable and could not result in acceptable P/AI. Therefore, ovulatory response to GnRH-1 is a key element for successful outcome of GnRH-based protocols (Bello et al., 2006; Colazo et al., 2015; Giordano et al., 2013; Vasconcelos et al., 1999). In this context, the ovulatory response of ovarian follicles to GnRH has been reported to improve by increase in follicle size (Sá Filho et al., 2010; Stevenson, 2025). This association is partly due to acquisition of more LH receptors and greater ovulatory capacity by larger follicles (Sartori et al., 2001; Xu et al., 1995). Furthermore, concentration of endogenous estradiol may contribute to this positive association between follicle size and ovulatory response to exogenous GnRH (Stevenson & Pulley, 2016). Because larger follicles produce higher estradiol (Perry et al., 2014; Vasconcelos et al., 2001), and higher estradiol concentration could promote effectiveness of exogenous GnRH in induction of a stronger LH surge (Macedo et al., 2021; Stevenson & Pulley, 2016). Therefore, the positive relationship between follicle size and its ovulatory response to GnRH could be one of the reasons for the positive association between size of LF at GnRH-1 and reproductive performance in the present study.

Nevertheless, extended interval between presynchronization and breeding protocols impaired reproductive performance of cows. Notably, two-day additional time between presynchronization and breeding protocols (i.e., O9DH and O9DH+PG groups) did not result in a significant change either in average size of LF or in proportion of cows in different categories of LF as compared with cows in O7DH group. This phenomenon implicates lack of progress in follicular growth during this additional two-day period, which might have led to prolonged dominance and aging of follicles (Abreu et al., 2014; Bleach et al., 2004). Prolongation of dominance and aging can diminish LH receptors and ovulatory capacity of follicles (Adams et al., 2008; Niribili et al., 2024; Xu et al., 1995). As aforementioned, successful ovulatory response to GnRH-1 plays a central role in efficiency of GnRH-based protocols (Bello et al., 2006; Chebel et al., 2006; Colazo et al., 2015; Giordano et al., 2013; Vasconcelos et al., 1999). As a result, the two-day extension might have compromised response to GnRH-1 by causing follicular aging, which further led to diminished fertility after TAI. Indeed, this finding implies that follicle size per se cannot be a determining factor for commencement of breeding protocol, and optimal size of follicle should be accompanied by optimal growing status of follicle to result in a favored outcome after breeding protocol.

The other hypothesis of this study concerning the effect of PGF_2α_ on PC was accepted since PGF_2α_-treated cows had lower PC than PGF_2α_-untreated ones. Likewise, Carvalho et al. (2015) studied the effect of PGF_2α_ adminstration two days before breeding Ovsynch in cows subjected to Double Ovsynch protocol, and found lower PC in PGF_2α_-treated cows as compared with their untreated counterparts. In this regard, PC as well as proportion of cows in various categories of PC were comparable between O7DH+PG and O9DH+PG groups. These findings implicate that day of administration (5 vs 7 days after presynchronization protocol) did not influence the effect of PGF_2α_ on induction of luteolysis in CLs. Yet extended interval in PGF_2α_-untreated cows (O7DH group vs O9DH group) led to an increase in PC and proportion of cows in PC3 category, and a decrease in proportion of cows in PC1 category. These findings might have been related to more development of CLs by progress in time after ovulation in response to the final GnRH of presynchronization protocol (Kelley et al., 2009).

Nevertheless, PC at GnRH-1 was not associated with P/AI and pregnancy loss. Similarly, Carvalho et al. (2015) observed comparable P/AI between cows with low and high PC at the beginning of breeding protocol. Conversely, there are other studies indicating that low PC at GnRH-1 could diminish P/AI in dairy cows (Bisinotto et al., 2013, 2015). Bisinotto et al. (2013, 2015) subjected cows to breeding Ovsynch, ultrasonographicaly scanned the ovaries of cows for presence of CL at GnRH-1, and treated a subset of cows without CL with controlled internal drug release (CIDR; for 7 days between GnRH-1 and PGF_2α_). They observed greater P/AI in CIDR-treated without-CL cows and with-CL cows compared with untreated without-CL cows (Bisinotto et al., 2013, 2015). However, it was not clear in these studies whether cows without CL were ovular and simply did not have CL at GnRH-1 or they were anovular and did not have CL even before the commnecment of breeding protocol (Bisinotto et al., 2013, 2015). Perhaps, association of PC at the commencement of breeding protocol with subsequent P/AI should be contextualized based on the cyclic status of cows (Stevenson & Lamb, 2016). If low PC at the initiation of breeding protocol is due to anovular ovaries, it could lead to inferior fertility (Bisinotto et al., 2010) because anovulation culminates in diminished oocyte and embryo quality (Santos et al., 2016). Moreover, the risk factors for anovulation (e.g., postpartum reproductive disorders and intensive negative energy balance) can have direct adverse effects on fertility of dairy cows (Santos et al., 2016). In other words, low P4 is not the cause of subfertility in anovular cows, and anovulation is the common cause for both low P4 and subfertility. Indeed, an anovulatory condition could be a lurking variable in studies conducted to understand the relationship between PC at GnRH-1 and fertility, potentially compromising the interpretation of the final results. In alignment with this concept, lower PC at the initiation of breeding protocol did not have such negative association with fertility in cyclic cows, and it even enhanced ovulatory response to GnRH-1 (Carvalho et al., 2015). Moreover, Bisinotto et al. (2022) revealed that lower P4 during follicular growth was associated with higher estradiol concentration before ovulation, ovulation of larger follicles and production of longer conceptuses; however, it was not strongly connected with transcriptome of conceptuses and endometrium. Therefore, initiation of GnRH-based protocols in cows with lower PC can have some positive impacts on effectiveness of GnRH-1 and the subsequent follicular wave, but may not significantly improve fertility of cows.

Furthermore, multiparous cows had larger follicles at GnRH-1 as compared with primiparous cows, which was in accord with the findings of previous studies (Keskin et al., 2016; Tenhagen et al., 2004). This effect of parity on follicle size has been attributed to level of milk production (Keskin et al., 2016; Tenhagen et al., 2004). Because higher milk production in multiparous cows cause greater hepatic metabolism of steroids, and in turn, lower circulatory concentration of these hormones, which could lead to development of larger follicles in multiparous cows (Keskin et al., 2016; Tenhagen et al., 2004). Nonetheless, level of milk production was not associated with follicle size in the current study. It is worth noting that level of milk production per se could not be the only determining factor regarding the effect of metabolic status on reproductive features of cows, and metabolic resilience of cows to different milk production levels may be a more important factor (Mobedi et al., 2024a, b; Wiltbank et al., 2006). In this sense, there is evidence indicating different metabolic function between primiparous and multiparous cows during postpartum period, including less lipolysis and loss of body condition score as well as greater glucose and insulin concentration in primiparous than multiparous cows (Gärtner et al., 2019; Hemmati Baghbanani et al. 2026; Wathes et al., 2007). In this study, however, we did not have data of metabolic status in the investigated cows to test whether the effect of parity on size of LF at GnRH-1 was related to metabolic mechanisms.

Moreover, P/AI was greater in primiparous than multiparous cows, which agreed with the findings of previous studies (Hemmati Baghbanani et al. 2026; Stevenson, 2025; Tenhagen et al., 2004). Interestingly, this greater fertility of primiparous cows was in spite of other observations of the current study, including smaller follicles at GnRH-1 in primiparous cows and the positive association of follicle size at GnRH-1 with P/AI. As mentioned above, the positive association between follicle size at the commencement of breeding protocol and fertility following TAI is complex and it could be affected by some covariates, and parity appears to be one of them.

Eventually it is worth mentioning that there were some limitations in the present research considering the large number of cows allocated to this study and the fact that it was conducted in commercial herds. In this regard, the managers of the herds gave us permission for only one ultrasonographic examination and blood sampling during the experiment in each cow. Therefore, we did not have data of ovarian status after GnRH-1 and before insemination. Moreover, we could not evaluate follicular persistence in cows with follicles larger than 20 mm in order to differentiate where they were cystic. Furthermore, it is noteworthy that although the results of this study did not completely support the proposed hypotheses, the findings still contribute to a better understanding of the response to these protocols of dairy cows and open opportunities for future research.

## Conclusion

In conclusion, extension in interval between presynchronization and breeding protocols led to no evident change in size of LF at GnRH-1; however, it diminished fertility of cows. Interestingly, size of LF at GnRH-1 was positively associated with reproductive performance. This finding implicates that modifications increasing size of LF at GnRH-1 could improve efficiency of GnRH-based protocols; however, extension between presynchronization and breeding protocols might not be an effective modification in this regard considering the results of this study. Furthermore, PGF_2α_ administration before breeding protocol could successfully reduce PC at GnRH-1, but it could not affect fertility of cows since PC at GnRH-1was not recognized as a determining factor on reproductive performance of cows subjected to TAI.

## Compliance with ethical standards

Animal Ethics Committee at University of Tehran approved the current research in terms of animal welfare and ethics (IR.UT.VETMED.REC.1403.054).

## Funding

This project was funded by Faculty of Veterinary Medicine, University of Tehran, Tehran, Iran (Grant No.: 30854/6/18).

## CRediT authorship contribution statement

**Reza Hemmati Baghbanani:** Writing – original draft, Methodology, Investigation, Data curation. **Emadeddin Mobedi:** Writing – original draft, Methodology, Investigation. **Amir Abbas Mohieddini:** Methodology, Investigation. **Poorya Pooladzadeh:** Methodology, Investigation. **Shayan Zarabadipour:** Methodology, Data curation. **Mehdi Vojgani:** Writing – review & editing, Supervision. **Faramarz Gharagozlou:** Writing – review & editing, Supervision. **Vahid Akbarinejad:** Writing – review & editing, Writing – original draft, Visualization, Supervision, Resources, Investigation, Formal analysis, Conceptualization.

## Declaration of competing interest

The authors have no conflict of interest to disclose.
